# Targeted expression profiling by RNA-Seq improves detection of cellular dynamics during pregnancy and identifies a role for T cells in term parturition

**DOI:** 10.1038/s41598-018-36649-w

**Published:** 2019-01-29

**Authors:** Adi L. Tarca, Roberto Romero, Zhonghui Xu, Nardhy Gomez-Lopez, Offer Erez, Chaur-Dong Hsu, Sonia S. Hassan, Vincent J. Carey

**Affiliations:** 1 Perinatology Research Branch, Division of Obstetrics and Maternal-Fetal Medicine, Division of Intramural Research, Eunice Kennedy Shriver National Institute of Child Health and Human Development, National Institutes of Health, U.S. Department of Health and Human Services (NICHD/NIH/DHHS), Bethesda, Maryland, and Detroit, Michigan USA; 2 Department of Obstetrics and Gynecology, Wayne State University School of Medicine, Detroit, Michigan USA; 3 Department of Computer Science, Wayne State University College of Engineering, Detroit, Michigan USA; 4 Department of Obstetrics and Gynecology, University of Michigan, Ann Arbor, Michigan USA; 5 Department of Epidemiology and Biostatistics, Michigan State University, East Lansing, Michigan USA; 6 Center for Molecular Medicine and Genetics, Wayne State University, Detroit, Michigan USA; 7 Channing Laboratory, Department of Medicine, Brigham and Women’s Hospital and Harvard Medical School, Boston, Massachusetts USA; 8 Department of Immunology, Microbiology and Biochemistry, Wayne State University School of Medicine, Detroit, Michigan, USA; 9 Department of Obstetrics and Gynecology, Soroka University Medical Center, School of Medicine, Faculty of Health Sciences, Ben-Gurion University of the Negev, Beer-sheba, Israel; 10 Department of Physiology, Wayne State University School of Medicine, Detroit, Michigan USA

## Abstract

Development of maternal blood transcriptomic markers to monitor placental function and risk of obstetrical complications throughout pregnancy requires accurate quantification of gene expression. Herein, we benchmark three state-of-the-art expression profiling techniques to assess in maternal circulation the expression of cell type-specific gene sets previously discovered by single-cell genomics studies of the placenta. We compared Affymetrix Human Transcriptome Arrays, Illumina RNA-Seq, and sequencing-based targeted expression profiling (DriverMap^TM^) to assess transcriptomic changes with gestational age and labor status at term, and tested 86 candidate genes by qRT-PCR. DriverMap identified twice as many significant genes (q < 0.1) than RNA-Seq and five times more than microarrays. The gap in the number of significant genes remained when testing only protein-coding genes detected by all platforms. qRT-PCR validation statistics (PPV and AUC) were high and similar among platforms, yet dynamic ranges were higher for sequencing based platforms than microarrays. DriverMap provided the strongest evidence for the association of B-cell and T-cell gene signatures with gestational age, while the T-cell expression was increased with spontaneous labor at term according to all three platforms. We concluded that sequencing-based techniques are more suitable to quantify whole-blood gene expression compared to microarrays, as they have an expanded dynamic range and identify more true positives. Targeted expression profiling achieved higher coverage of protein-coding genes with fewer total sequenced reads, and it is especially suited to track cell type-specific signatures discovered in the placenta. The T-cell gene expression signature was increased in women who underwent spontaneous labor at term, mimicking immunological processes at the maternal-fetal interface and placenta.

## Introduction

Human blood is a rich source of molecular information that can be used to develop non-invasive liquid biopsies for specific tumors^1^ and organs (e.g., the placenta)^2^ in order to predict disease, its progression, and response to treatments. Genome-wide transcriptomic profiling is particularly well-suited for the discovery of molecular markers^3^ given the availability of techniques such as microarrays^4^ and RNA sequencing (RNA-Seq)^5,6^, which allow simultaneous measurement of tens of thousands of protein-coding and non-coding genes in a given sample.

Cellular and cell-free RNAs in blood that originate from (or are specific to) the primary tumor or organ of interest are especially sought as candidate biomarkers^2,7,8^ and, more recently, owing to advances in single-cell genomics^9^, researchers developed cell type-specific signatures of tissues, e.g., the placenta. This approach holds the promise to unravel the complexity of the maternal-fetal molecular dialogue^2^ and to aid in developing liquid biopsies for prediction of the ‘great obstetrical syndromes’^10^. Since organ and/or cell type-specific transcripts are expected to have low expression in whole blood, it is essential that quantification of RNA abundance is accurate enough so that modest, and eventually coordinated, gene expression changes can be leveraged as biomarkers that have clinical utility.

The complexity of quantifying low-abundance RNAs using conventional microarrays and RNA-Seq is compounded by the presence of high and variable levels of globin mRNA and ribosomal RNA (rRNA). Although rRNA depletion and globin reduction have been shown to mitigate some of these issues, they require a large amount of total RNA and may induce biases in the quantification of gene expression^11^. To address these limitations, targeted expression profiling methods were developed based on multiplex RT-PCR amplification followed by quantitative analysis of mRNA abundance by next-generation sequencing. Although microarrays^12–15^ and RNA-Seq^6,16,17^ were previously benchmarked for differential expression and prediction model development, preprocessing methods for RNA-Seq have continued to evolve^17,18^, and direct comparisons to targeted expression profiling by RNA-Seq for genome-wide transcription are not available.

Therefore, the goal of this work was to compare Affymetrix Human Transcriptome Arrays (HTA 2.0) that probe the transcriptome at the exon level, paired-end Illumina RNA-Seq with globin reduction, and a novel genome-wide targeted expression profiling technique DriverMap (https://www.cellecta.com). The comparison was made in terms of ability to identify true maternal whole blood expression changes with gestational age and with the onset of labor at term. Furthermore, we have for the first time evaluated the ability of these high-throughput methods to quantify in maternal whole blood the expression of cell type-specific signatures derived from single-cell genomics of the placenta, and we also have determined whether these signatures are indicative of the onset of term parturition.

## Results

RNA was extracted from 32 maternal whole blood samples from women with a normal pregnancy with (n = 8) and without (n = 8) spontaneous labor at term. The two groups will be further referred to as term in labor (TIL) and term not in labor (TNL). One-half of the women in each group had three longitudinal samples taken at 12 to 40 weeks of gestation, while the other one-half of the women had one sample taken at term before delivery (see Table S1). The RNA integrity number (RIN) was very similar among samples (range 6.2 to 7.4) and did not change with sample storage duration (p = 0.6). Data generated from three high-throughput gene expression platforms were made available to the community as a Gene Expression Omnibus super series (https://www.ncbi.nlm.nih.gov/geo/query/acc.cgi?acc=GSE113966) and may be valuable in future studies assessing RNA-Seq quantification workflows.

### Detection of transcripts in maternal whole blood

Affymetrix Human Transcriptome Arrays (HTA 2.0) probed 30,682 annotated transcript clusters, of which 24,371 coding and 5,389 non-coding transcript clusters were deemed expressed in maternal whole blood. Paired-end Illumina RNA-Seq generated 20.7 to 57.1 million aligned sequence fragments per sample (mean, 39.4 million), enabling detection of 32,880 genes, of which 15,584 were protein-coding genes. When limited only to the 18,559 protein-coding genes targeted by DriverMap, the number of aligned reads per sample obtained for RNA-Seq was 2.6 to 6.6 million (mean, 4.6 million). Targeted profiling of 18,559 protein-coding genes by DriverMap resulted in 11.1 to 12.9 million aligned sequence fragments per sample (mean, 12.0 million), allowing detection of 13,182 genes. Expression profiling for 1/32 samples by DriverMap was not successful due to contamination, and it was not included in downstream analyses.

### Differential expression associated with gestation and term labor

**Figure 1:**
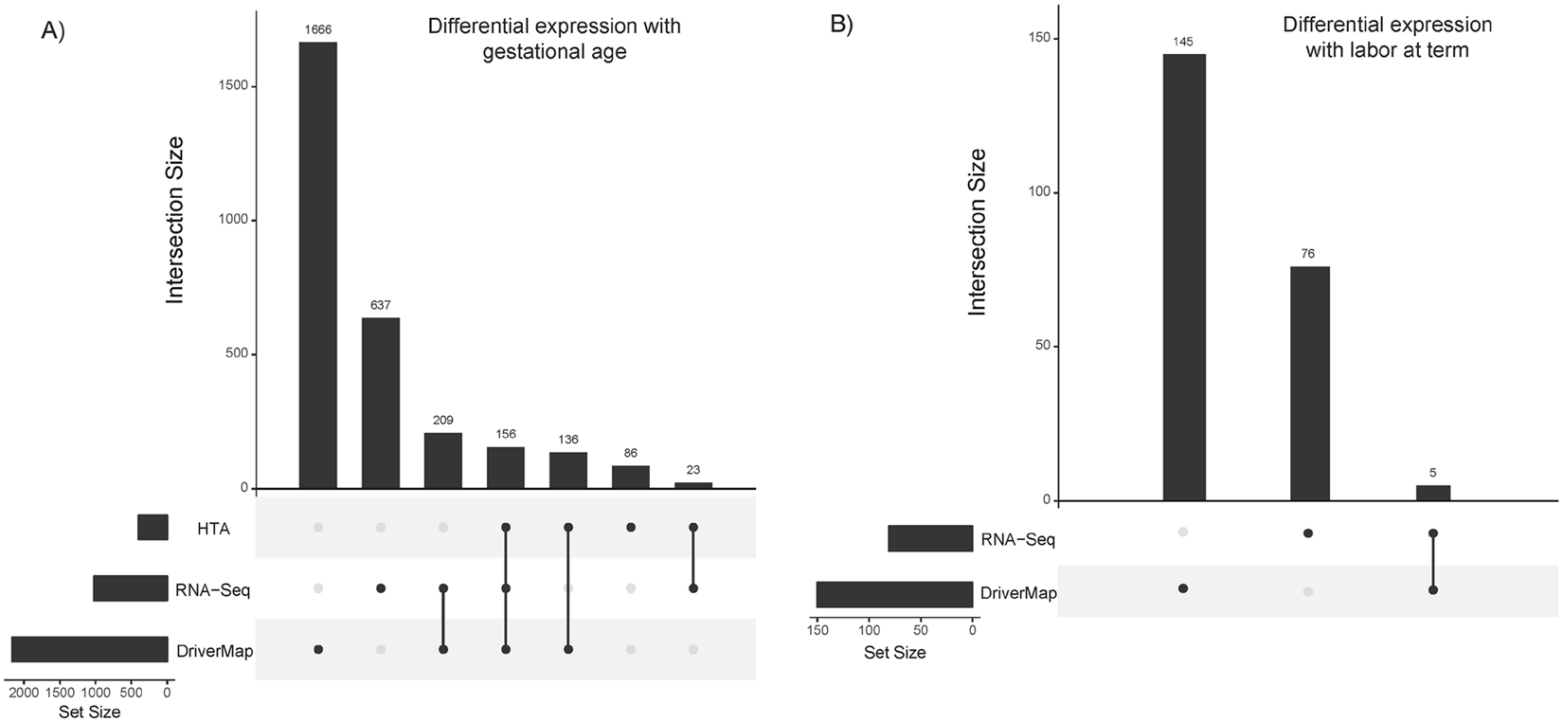
UpSet plots of genes differentially expressed using three transcriptomic platforms. Changes with gestational age (term vs preterm) (left) and with labor status (term in labor vs term not in labor) (right). The significance of gene expression changes was inferred based on an adjusted p-value (q-value) <0.1. The horizontal bars show the number of differentially expressed genes identified by each method, while the vertical bars display the size of sets of genes identified by only one method and the intersection sets.

The UpSet plots^19^ in Fig. 1 summarize the overlap of gene expression changes associated with gestational age (term vs preterm gestation) (Fig. 1A) and with labor at term (Fig. 1B) for the three high-throughput transcriptomic platforms. At the same significance cut-off (false discovery rate^20^, q < 0.1), 401, 1025, and 2167 genes were found to change with gestation by microarrays, RNA-Seq, and DriverMap, respectively. Of note, 156 differentially expressed genes were identified by all three platforms to change in the same direction, representing 40% of the size of the smallest of the three lists (Fig. 1A). Fewer genes were found to change with labor at term, with only RNA-Seq and DriverMap identifying 81 and 150 genes, respectively, of which only five were in common (GZMB, KLRC1, CD69, and KLRF1 were increased while SPTB was decreased with labor) (Fig. 1B).

### qRT-PCR validation of gene expression changes

qRT-PCR TaqMan assays (Applied Biosystems, Foster City, CA) were used to profile 86 candidate genes in the same 32 samples, with genes being selected based on evidence for differential expression with either gestational age or labor according to one or more platforms (see *Methods*). Of note, the average expression over the six house-keeping genes was stable with sample storage duration (linear correlation p = 0.87, Fig. S1). When using qRT-PCR results to define true positive changes (two-tailed moderated t-test, p < 0.05) (see Tables S2 and S3), the validation rate of changes with gestation was 98% (40/41) for microarrays, 94% (33/35) for RNA-Seq, and 88% (44/50) for DriverMap. Of note, although the list of genes included in the calculation of validation rates (i.e. positive predicted values) for each platform represent a different subset of all 86 genes profiled by qRT-PCR, the ranks of validation genes among the list of differentially expressed genes (positive genes) were not significantly different among platforms (ANOVA p = 0.15) (Fig. S2). The validation rate for changes with onset of labor at term was 45% (5/11) for RNA-Seq and 96% (23/24) for DriverMap, and it could not be determined for microarrays given that no gene was significant at q < 0.1. The lower validation rate for RNA-Seq for changes with labor compared to changes with gestation was expected, since for the former condition the genes were selected from the bottom half while in the latter from the upper half of the list of genes ranked by p-values (Figs S2 and S3).

**Figure 2:**
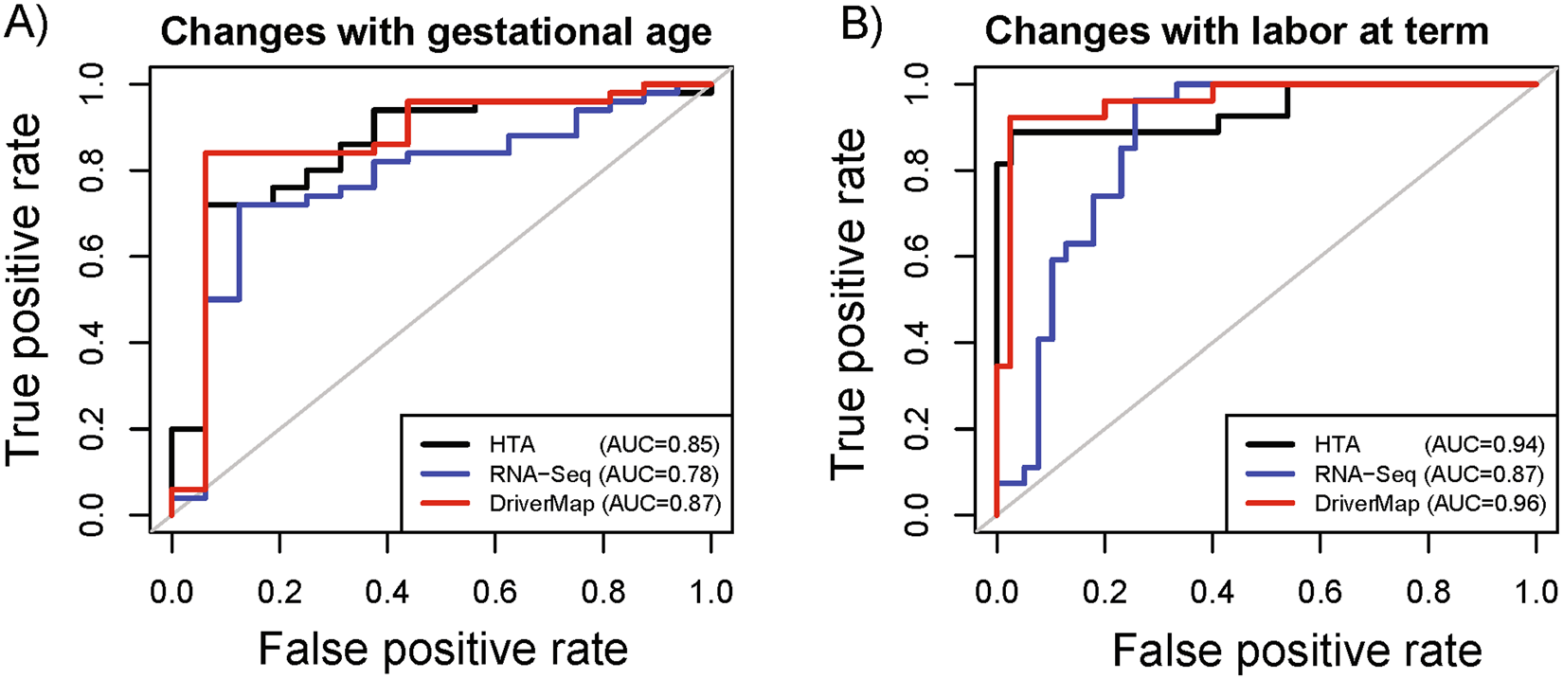
Receiver operating characteristic (ROC) curves for detection of differentially expressed genes. Of the 86 genes profiled by qRT-PCR, 66 were deemed detected by all three platforms and were deemed truly differentially expressed with gestational age (**A**) and with labor at term (**B**) if significant by qRT-PCR analysis. HTA: Human Transcriptome Arrays; AUC: area under the curve.

In addition to qRT-PCR validation rates, which can be seen as estimates of positive predicted values (PPV) for each platform (rate of true differentially expressed genes among all genes positives at q-value < 0.1), we have also compared the area under the receiver operating characteristic curve (AUC) among the three platforms based on 66 protein-coding genes. These 66 genes were all of the 86 genes selected for qRT-PCR validation (see *Methods* section) that were detected present on all three platforms (Fig. 2). To construct the ROC curves for a given platform, the 66 genes were ranked by differential expression p-values obtained with the particular platform. The AUC values for expression changes with gestational age were 0.78, 0.85 and 0.87 for RNA-Seq, HTA, and DriverMAp, respectively. Similarly, for changes with labor at term, the AUC statistics were 0.87, 0.94 and 0.96 for RNA-Seq, HTA, and DriverMap, respectively. Of note, although no gene was differentially expressed with labor based on the HTA platform after multiple testing correction, the ability of this platform to rank truly differentially expressed genes at the top of the list is only slightly lower than the one of DriverMap and surpasses then one of RNA-Seq (Fig. 2B).

### Correlation of expression changes between high-throughput methods and qRT-PCR

**Figure 3:**
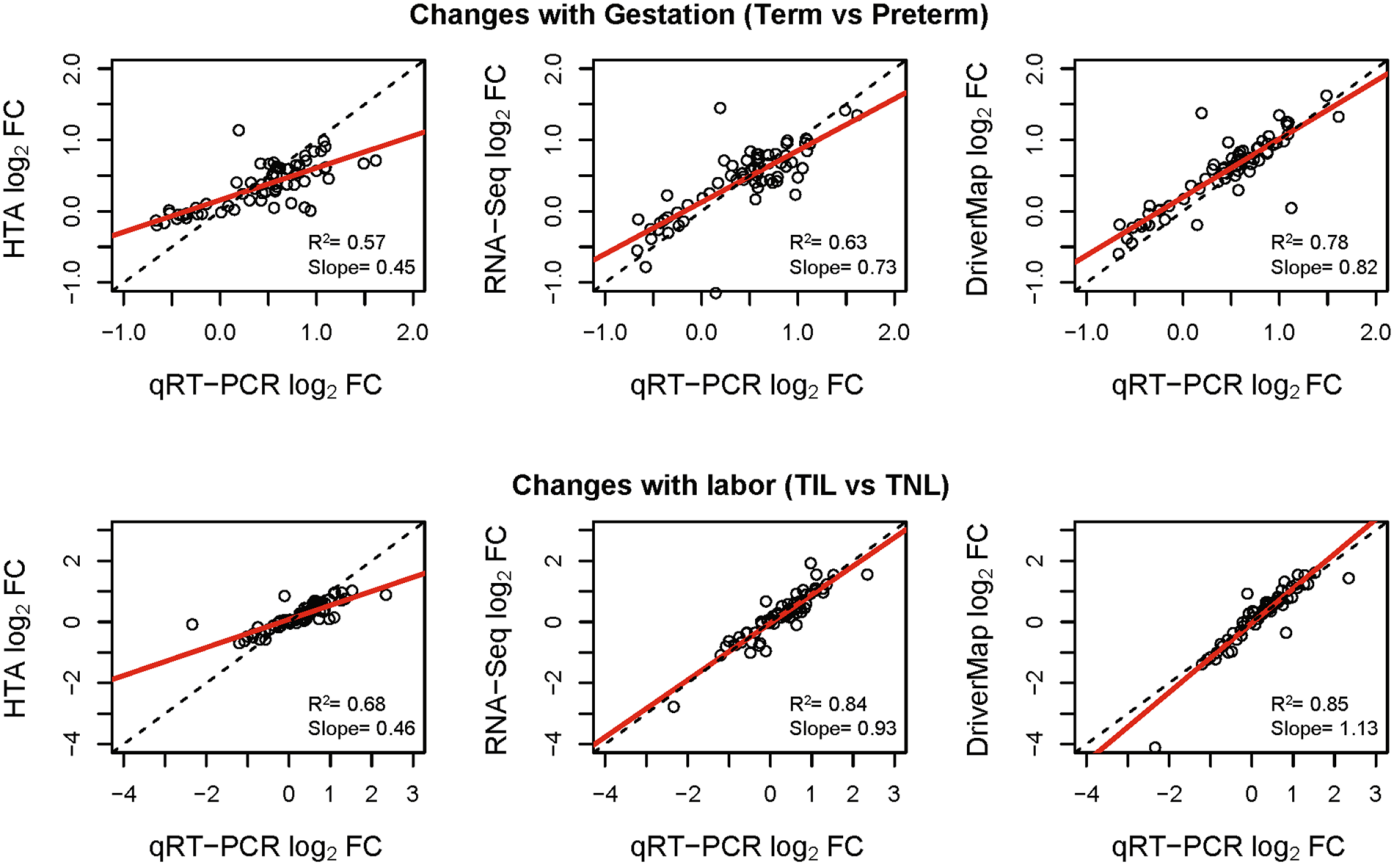
Correlation of expression changes between high-throughput platforms and qRT-PCR. Of the 86 genes profiled by qRT-PCR, 66 were deemed detected by all three platforms and are displayed as individual dots in this figure. TIL: term in labor; TNL: term not in labor; HTA: Human Transcriptome Arrays; FC: fold change.

We have conducted a correlation analysis to determine the agreement in the estimated gene expression fold changes (log_2_ thereof) obtained with high-throughput expression profiling techniques and qRT-PCR. The correlation was based on estimates of log_2_ fold changes obtained for 66 genes detected present, but not necessarily found significant, with all three platforms. The linear regression slope (95% confidence intervals) between high-throughput methods and qRT-PCR log_2_ fold-changes was 0.45(0.35–0.55) and 0.46(0.38–0.53) for microarrays, 0.73(0.59–0.86) and 0.93(0.83–1.03) for RNA-Seq, and 0.82(0.71–0.92) and 1.13(1.01–1.25) for DriverMap for changes with gestation (Fig. 3 top) and with labor (Fig. 3 bottom), respectively. Slope estimates were significantly higher for the two sequencing-based methods than for microarrays, yet confidence intervals overlapped between RNA-Seq and Drivermap. In this analysis, slopes <1.0 correspond to a compression, while slopes >1.0 correspond to an expansion of the dynamic range of expression changes compared to qRT-PCR. For instance, the 0.46 slope estimate obtained for microarrays can be interpreted that, in average, a gene showing a 2-fold change in expression by qRT-PCR between TIL and TNL groups, displays a 1.37 fold change with microarrays; however, the corresponding fold changes were 1.93 with RNA-Seq and 2.2 fold with DriverMap (Fig. 3 bottom).

### Comparison to other differential expression studies

The lists of genes associated with gestational age by each high-throughput platform were overlapped with those reported in previous studies^21,22^. Heng *et al*.^21^ reported paired maternal whole blood gene expression changes from 17–23 weeks to 27–33 weeks of gestation in 114 women with term delivery. The authors used Affymetrix Human Gene 2.1 ST microarrays and found 41 genes up-regulated and four down-regulated at q < 0.05 and fold-change >1.25. To increase the power for testing for an overlap between the list of Heng *et al*. and this study, we have included all 2,321 genes significant at q < 0.05. Another longitudinal study by Al-Garawi *et al*.^22^, using Illumina HumanHT-12 v4 microarrays, reported dramatic changes in the transcriptome from 10–18 weeks to 30–38 weeks of gestation in 30 women included in a trial of vitamin D supplementation. The authors found expression changes associated with gestation in 3,830 unique genes at q < 0.05. To enable a direct comparison between the different transcriptomic platforms, enrichment analyses were limited to genes profiled on all three platforms. We found a significant enrichment in previously reported differential expression associated with gestational age among genes identified by each of the three platforms used herein (odds ratios, OR of 1.4–2.1; hypergeometric test p < 0.01 for all) (Table 1). Enrichment statistics (ORs) were higher for microarrays, yet DriverMap identified a larger number of genes previously reported to change with gestation.

**Table 1:** Enrichment analysis of previously reported changes with gestational age among the results of this study.

Dataset	Platform	Common significant genes (N)	Odds Ratio	p-value
Heng *et al*.	DriverMap	335	1.4	2.7E-06
Heng *et al*.	HTA	73	1.7	7.5E-05
Heng *et al*.	RNA-Seq	97	1.4	2.0E-03
Al-Garawi *et al*.	DriverMap	711	1.8	3.5E-31
Al-Garawi *et al*.	HTA	142	2.1	5.8E-11
Al-Garawi *et al*.	RNA-Seq	198	1.8	8.6E-10

### Quantification of cell type-specific signatures discovered by single-cell genomics

**Figure 4:**
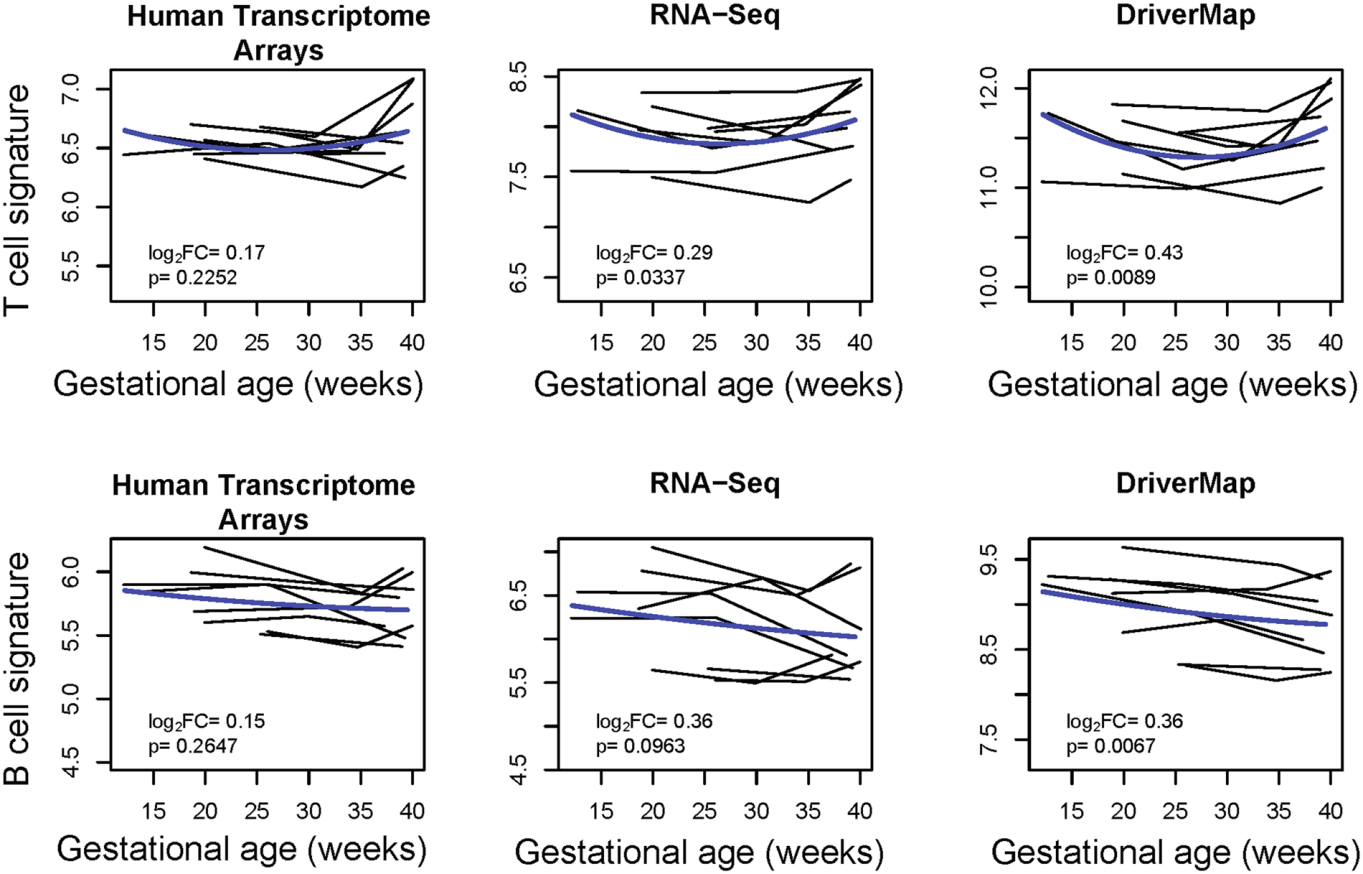
Changes in average expression of cell type-specific genes with gestational age. Gene expression for 17 T-cell-specific genes (top) and 12 B-cell-specific genes were averaged and displayed (y-axis) as a function of gestational age at sampling (x-axis). For microarrays, averages are over log_2_ normalized expression intensity. For sequencing based techniques, the average is over log_2_ *DESeq2* normalized count data. Each line corresponds to one woman. The blue line represents a linear mixed-effect model fitted by using quadratic splines with one knot.

We compared the ability of the three high-throughput platforms to quantify in maternal whole blood the abundance of cell type-specific gene sets discovered by single-cell transcriptomics of the placenta^2^. In this analysis, 11 cell type-specific gene signatures for which three or more genes were detected by all three platforms were included. According to Tsang *et al*.^2^, the B-cell-specific signature decreases monotonically as a function of gestation, while the T-cell-specific signature decreases from the first to second trimester and then increases during the third trimester. Figure 4 shows that, indeed, a significant quadratic u-shaped trend was found by linear mixed-effects models for T-cell expression quantified by RNA-Seq and DriverMap, and a significant linear decreasing trend for B cell expression quantified by DriverMap (all p < 0.05). Although DriverMap data led to stronger associations with gestational age for these signatures (smaller p-values) the confidence intervals of the log_2_ fold changes overlapped among methods.

**Figure 5:**
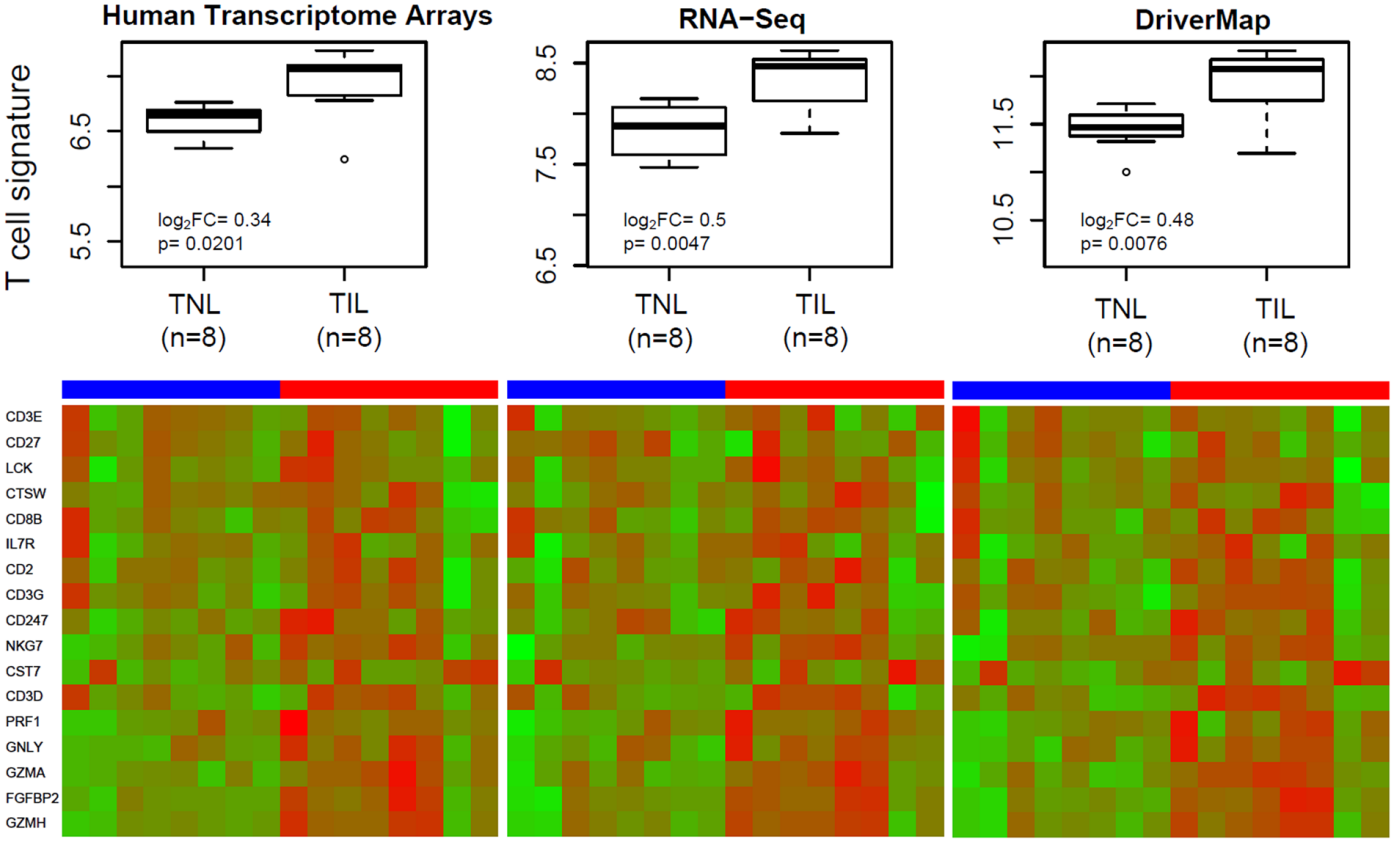
Changes of T-cell-specific gene signature with labor status in maternal whole blood. Gene expression for 17 T-cell-specific genes was summarized in each sample collected at term from women in labor (TIL) and not in labor (TNL). Expression levels of individual genes are also shown using a heatmap.

When the cell type-specific gene signatures were tested for the first time for association with the onset of labor at term, we found that the average expression of genes in the T-cell signature, comprised of 17 genes, was higher in women who were in labor compared to those who were not in labor at the time of the blood draw (Fig. 5). The fold-change estimates were higher for RNA-Seq and DriverMap compared to microarrays, yet 95% confidence intervals overlapped [log2 fold change 0.34(0.08, 0.60) for microarrays, 0.5(0.18, 0.81) for RNA-Seq and 0.48(0.16, 0.81) for DriverMap]. The increase of T-cell-specific gene expression with labor at term was also confirmed by qRT-PCR profiling of 4/17 genes (*GZMH*, *GNLY*, *FGFBP2*, and *GZMA*) (p = 0.0048, 2.5 fold-change).

**Figure 6:**
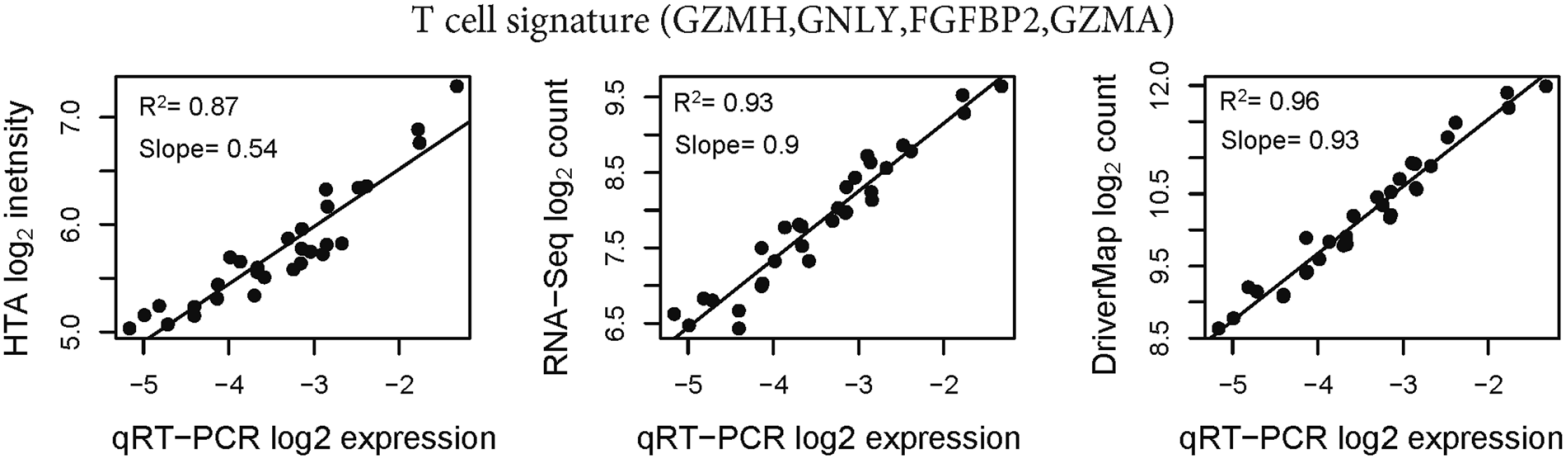
Correlation analysis of T-cell-specific gene signatures between high-throughput methods and qRT-PCR. The x-axis shows qRT-PCR expression averages (−ΔCt) over *GZMH*, *GNLY*, *FGFBP2*, and *GZMA* genes in individual samples, while the y-axis shows the same summaries derived from high-throughput expression profiling methods.

When expression of the T-cell signature was summarized based on four genes (*GZMH*, *GNLY*, *FGFBP2*, and *GZMA*) for which qRT-PCR data were also available, the correlation with qRT-PCR expression and dynamic range of the T-cell signature average expression across samples were slightly higher for DriverMap than RNA-Seq, and both sequencing-based methods performed better than microarrays (Fig. 6).

## Discussion

### Detection of mRNAs in maternal whole blood

The number of protein-coding genes detected as present in maternal whole blood samples was 24,371, 15,584, and 13,182 for Human Transcriptome Arrays, RNA-Seq, and targeted expression profiling by DriverMap, respectively. Differences in the number of genes detected by microarrays and sequencing techniques in the same samples are to be expected due to platform design differences, experimental differences, level of background noise, and criteria used to determine expression above background^16,23,24^. While using detection p-values to infer expression above background for microarrays has been widely adopted^25,26^, a minimum number of aligned sequence fragments is typically required for RNA-seq (e.g., ≥10^27^, ≥20^28^), with the stringency of these cut-offs being dependent on the sequencing depth. Our choice of a minimum transcript count of 5 to call a gene detected in a given sample, for which an average 40 million aligned reads were obtained (5/40 = 0.125 transcripts per million [TPM]) is similar to the 0.1 TPM cut-off used in a recent benchmark of RNA-sequencing analysis workflows^24^. Another important factor contributing to the differences in the number of genes detected by sequencing methods is the depth of coverage^29^. Although over three times more total aligned reads per sample were obtained for RNA-Seq than for DriverMap, the number of aligned reads per sample for protein-coding gene content was about three times less for RNA-Seq and it varied more across samples compared to DriverMap. Of note, both the HTA microarrays and RNA-Seq probed also non-coding RNAs, and small RNAs that have compelling biological and disease roles^30^, including in pregnancy^31–34^.

### Gene expression changes associated with gestation and with labor at term

Longitudinal transcriptomic changes associated with gestational age (term vs preterm gestation), and cross-sectional changes associated with labor at term (term in labor vs term not in labor), were assessed using commonly used analytical approaches that borrow information across genes to derive more reliable expression variance estimates^35,36^. HTA microarrays identified 2.6 times fewer differentially expressed genes than RNA-Seq and 5.4 times fewer than DriverMap at the same false discovery rate (q < 0.1). When a more conservative false discovery rate cut-off was used (q < 0.05), HTA microarrays identified 3.7 times fewer differentially expressed genes than RNA-Seq and 8.6 times fewer than DriverMap (Fig. S4). Although differences in the type of data (continuous intensity for microarray vs read counts for sequencing methods) and, hence, analysis models may be a factor, the higher background noise^37,38^ resulting in compressed fold-changes with microarray data (Fig. 3) and the more stringent correction required to maintain the same false discovery rate explain, in part, these results. Indeed, when only the genes deemed to be expressed on all three platforms were tested, the number of genes differentially expressed (q < 0.1) with gestation by HTA increased from 401 to 636, narrowing the gap with RNA-Seq while still being more than three times lower than the one for DriverMap (Fig. S5). Our finding that Illumina RNA-Seq identifies more differentially expressed genes compared to microarrays is in line with previous reports^39^, although those studies used 3′-end biased microarrays as opposed to arrays that probe all exons of the genes, as it was the case herein.

### qRT-PCR validation of gene expression changes

Although profiling by DriverMap identified more differentially expressed genes than microarrays and RNA-Seq, the qRT-PCR validation rate (i.e., the fraction of differentially expressed genes confirmed by qRT-PCR among all differentially expressed genes that were tested) was about the same for this platform (92% overall for changes with gestation and with labor) compared to the other two platforms (98% for microarrays and 94% for RNA-Seq for changes with gestation). Of note, for changes with labor, the validation rate of microarrays could not be assessed given that no positive genes were found, while the lower validation rate (45%) for RNA-Seq for changes with labor compared to the one for changes with gestational age was expected due to differences in the ranks of genes tested by qRT-PCR among those differentially expressed (see Figs S2 and S3). It is important that the ranks of genes tested are similar when comparing validation rates among platforms, as one would expect that genes with the smallest p-values (higher ranks) are more likely to be truly differentially expressed than those that appear lower on the list; indeed, this was the case for validation of RNA-Seq results.

While the comparison of validation rates (or PPV) required the use of a significance cut-off to define a positive result (i.e. q < 0.1), the threshold free AUC statistics revealed that the ranking of genes based on nominal p-values is similarly meaningful for microarrays and DriverMap platforms and somewhat lower for RNA-Seq, yet 95% confidence intervals of AUC statistics overlapped among platforms.

The correlation of fold changes derived from high-throughput platforms and qRT-PCR (reference method) were higher for sequencing methods than microarrays and especially for DriverMap (Fig. 3). The estimated R^2^ coefficient for correlation of Salmon^40^ quantified RNA-Seq expression and qRT-PCR expression changes in a two group analysis (TIL vs TNL) was identical to the one previously reported (0.85) based on wet-lab validation of 18,080 protein-coding genes in human brain tissues^24^ and relying on RNA-Seq data generated in the FDA sponsored Sequencing Quality Control (SEQC) study^12,16^. The more compressed fold-changes derived from microarrays than with sequencing based techniques was expected due to the background levels owing to cross-hybridization^37,38^. Of interest, for changes associated with labor at term, the dynamic range of DriverMap exceeded the one of qRT-PCR, with the slope in the log_2_ fold change correlation plot significantly exceeding parity (slope of 1.13, which is significantly >1.0).

### Comparisons to other studies of transcriptomic changes with gestation

The impact of advancing gestation on the maternal whole blood transcriptome in normal pregnancies has been previously evaluated, and results vary depending on the intervals of gestation considered, sample size, expression profiling platforms, and significance cut-offs. Heng *et al*.^21^ reported only 45 genes changing with gestation from 17–23 to 27–33 weeks. This number increases to 2,321 if the cut-off on the magnitude of change is removed and hence genes are selected based only on adjusted p-values. Al-Garawi *et al*.^22^ reported that 12% of the transcriptome (3,830 unique genes) is modulated during gestation, yet in this later study, the span of gestation was much larger, including first-trimester and term gestation samples (10–18 weeks vs 30–38 weeks). This larger fraction of genes changing with gestation is rather similar to the one of maternal plasma proteins that we have previously reported to change with gestational age, using frequent sampling from early to term gestation^41^. All three transcriptomics platforms evaluated in this study identified significantly more genes than expected by chance among those reported previously. Although enrichment ORs were higher for the HTA microarrays, DriverMap confirmed more of the previously reported gene changes and provided the strongest evidence for an enrichment (smallest p-values) (Table 1).

The importance of identifying transcripts associated with gestational age was recently highlighted in a study by Ngo *et al*.^8^ who screened cell free RNA transcripts among those that are placental, immune, and fetal-liver specific, with the goal of developing a prediction model for the interval from blood draw to term delivery. Of the 20 immune and placenta-specific genes highlighted as changing with gestation by Ngo *et al*.^8^ and also detected on all three platforms in maternal whole blood, DriverMap identified five (ANXA3, ARG1, S100A8, S100P, and ADAM12) as being significantly modulated during gestation, followed by HTA microarrays and RNA-Seq that identified three and one of these genes, respectively.

### Cell type-specific gene expression signatures as markers for placental function

Gene set analysis is one of the few means available to researchers to interpret omics studies and translate omics findings across platforms and species^14,42,43^. When the number of differentially expressed genes identified in a given condition is large, gene set analysis can be used to identify a few categories of genes that share a similar function and that are over-represented/enriched within the list of differentially expressed genes^44^. By contrast, when no significant changes can be demonstrated at the gene level, for example, due to a modest effect or small sample size, gene set analysis can use modest but coordinated changes in expression to establish a link between the phenotype and a predefined group of functionally related genes^45^. Another approach that implements gene set information to increase statistical power is the use of a gene set level summary as a biomarker^46^. In pregnancy research, Tsang *et al*.^2^ characterized the expression patterns of subpopulations of placental cells, including some of fetal (e.g., extravillous trophoblast and syncytiotrophoblast), maternal (e.g., decidual cells) and mixed origin (e.g., T cells). The authors have defined cell type-specific genes as those having higher expression in a given cell type compared to all others in the population of cells, similar in concept to defining tissue-specific gene sets^47,48^. The cell-free RNA expression (normalized sequence count) average over genes in the extravillous trophoblast signature was shown to be elevated in plasma of women diagnosed with preeclampsia compared to those with normal pregnancy. The authors have also shown that, of all cell-type signatures considered, the expression of B-cell and T-cell signatures changed most markedly with gestational age. While B-cell signature decreased monotonically from the first to the third trimester, T-cell signature decreases from the first to the second trimester and then increased during the third trimester. Despite differences between these two studies in terms of the type of samples (plasma vs whole blood) and downstream methods for RNA preservation and separation/extraction, our data strongly support these expression patterns with gestational age. A possible explanation for these similarities is that the plasma cell-free RNA included transcripts released by the white blood cells (cellular transcriptome reported herein). We also demonstrated that targeted expression profiling by DriverMap is especially suitable to track cell type-specific signatures as it leads to stronger associations and expression summaries that correlate better with qRT-PCR results (Figs 4 and 5).

### T-cell signature as a marker for onset of labor

This work demonstrated for the first time a significant increase in the T-cell expression signature in maternal whole blood from women who underwent spontaneous labor at term compared to those who delivered at term without labor. This observation is in line with evidence supporting a role for maternal T cells in the physiologic and pathologic processes of term and preterm labor, including: (1) effector and activated T cells are found at the maternal-fetal interface before^49–55^ and during the physiologic process of labor^56–58^; (2) effector T cells are present in the peripheral blood before^59–61^ and during labor^62^; (3) the absence of T cells results in increased susceptibility to endotoxin-induced preterm labor, which was reversed by the adoptive transfer of CD4^+^ T cells^63^; (4) histopathological lesions characterized by the infiltration of maternal T cells into the placental tissues (i.e., villitis of unknown etiology^64–66^, chronic chorioamnionitis^67^, and chronic deciduitis^68^) are associated with preterm labor and labor at term^67,69–73^; (5) effector and regulatory T-cell subsets at the maternal-fetal interface are associated with the timing of term parturition^74^ and the onset of preterm labor^75–77^; and (6) *in vivo* T-cell activation causes the onset of preterm labor^78^. More recently, we reported that fetal T cells can also participate in the mechanisms that lead to spontaneous preterm labor by responding toward maternal antigens and releasing pro-inflammatory cytokines^79^. Collectively, these data indicate that T cells are implicated in the physiological and pathological processes of labor and that sequencing-based techniques that quantify cell type-specific signatures in maternal whole blood could provide a read-out of the immunological events taking place at the maternal-fetal interface and in the placenta.

## Conclusions

Sequencing-based techniques are more suitable to quantify whole blood gene expression compared to microarrays, as they have an expanded dynamic range and identify more true positives. Targeted expression profiling by DriverMap identified more differentially expressed genes with gestational age and with labor at term than the other two platforms, and it is more accurate than untargeted RNA-Seq in measuring protein-coding gene expression, as it achieves higher coverage for coding genes for a lower total number of sequenced reads per sample. We have also demonstrated that targeted expression profiling is especially suited to track cell type-specific signatures discovered by single-cell transcriptomics in placental tissues and showed that maternal whole blood could provide a readout of the immunological events taking place at the maternal-fetal interface and in the placenta.

## Methods

### Study design

The study was part of a prospective longitudinal study that enrolled women with a normal pregnancy attending the Center for Advanced Obstetrical Care and Research of the Perinatology Research Branch, NICHD/NIH/DHHS, and the Detroit Medical Center/Wayne State University from February 2011 to May 2015. For gene expression profiling, we selected normal pregnancies with (n = 8) and without (n = 8) spontaneous labor at term. For one-half of the women in each labor group, we profiled three longitudinal samples collected at 12 to 40 weeks of gestation (Fig. 4), while for the other one-half of the women in each labor group, we profiled one sample taken at term before delivery (Fig. 5), for a total of 32 transcriptomes (Table S1). All patients provided written informed consent. The use of biological specimens as well as clinical data for research purposes was approved by the Institutional Review Boards of Wayne State University and NICHD. All experiments were performed in accordance with relevant guidelines and regulations.

### Sample collection and processing

Whole blood samples were collected directly into PAXgene Blood RNA tubes, stored at room temperature for 24 hours, and frozen at −80 °C until analysis. The PAXgene tubes contain a stabilizing additive to maintain cellular RNA integrity for extended periods of time; cellular RNA integrity is reported to be preserved up to 5 years^80^.

### RNA Extraction

RNA was isolated in April 2016 from PAXgene® Blood RNA collection tubes (BD 762165), as described in the PAXgene® Blood miRNA Kit Handbook (December, 2015). Purified RNA was quantified by UV spectrophotometry using the DropSense96® Microplate Spectrophotometer (Trinean) and quality assessed by microfluidics using the RNA ScreenTape on the Agilent 2200 TapeStation.

### Microarray profiling

A quantity of 100 ng of RNA was reverse-transcribed and amplified using the Affymetrix WT Plus Expression Kit (needs vendor information), following the manufacturer’s suggested protocol. A quantity of 5.5 μg of sense strand cDNA was fragmented and labeled using the Affymetrix WT Terminal Labeling Kit. 200 μl of labeled targets were hybridized to Affymetrix Human Transcriptome Arrays 2.0 GeneChip in an Affymetrix hybridization oven at 45 °C at 60 rpm for 16 hours. Washing and staining were performed on an Affymetrix Fluidics Station 450 and scanned on an Affymetrix GeneChip scanner 3000. Raw intensity data were generated from array images using Affymetrix AGCC software.

### RNA-Seq profiling

Starting with 500 ng of total RNA, cDNA library templates were synthesized using the Illumina TruSeq® Stranded Total RNA LT (Set A) Kit with Ribo-Zero Globin rRNA reduction, as described in the TruSeq® Stranded Total RNA Sample Preparation Guide (Rev. E., October 2013). Libraries were validated using the HS D1000 ScreenTape on the Agilent 2200 TapeStation and quantified on the Qubit® 2.0 Fluorometer by the Qubit® dsDNA HS Assay (ThermoFisher). The Illumina HiSeq2500 instrument was used to cluster the samples onto the flow cell. The pools were put on at 10 pM and run in rapid mode at 2 × 100 paired end. The Illumina HiSeq Rapid PE Cluster Kit V2 and HiSeq Rapid SBS Kit v2 200 cycles were used.

### Targeted expression profiling by DriverMap

The DriverMap Human Genome-Wide Gene Expression Profiling Assay (hDM18Kv2; Cellecta Inc., Mountain View, CA) was used to measure the expression level of 18,559 protein coding genes by combining highly multiplexed RT-PCR amplification with Next-Generation Sequencing quantitation. Sample processing was performed per manufacturer protocols available at https://www.cellecta.com/technology-portfolio/targeted-expression-profiling-driver-map-assay/. cDNA products amplified in the assay were analyzed on an Illumina NextSeq. 500 sequencer using a NextSeq500/550 High Output v2 Kit (75 cycles).

### qRT-PCR profiling

Total RNA (150 ng/sample) was reverse-transcribed into cDNA using Reverse Transcription Master Mix (100–6298, Fluidigm, San Francisco, CA). The reaction system included 150 ng of sample total RNA and 1 μl of the master mix plus RNase-free water to bring the final volume to 5 μl. The BioMark System (Fluidigm) was used to perform high-throughput qPCR. For this system, specific target amplification of cDNA was performed. Briefly, a 0.2X pool of specific TaqMan gene expression assays (Applied Biosystems) was prepared by mixing the individual 20X assays (total 93, 1 μl of each) and 7 μl of RNase-free water and used as the source of primers. Pre-amplification reactions contained 1.25 μl of cDNA, 2.5 μl of TaqMan PreAmp Master Mix (4391128, Applied Biosystems), and 1.25 μl of the pooled TaqMan assay mix. The reaction was performed using the 7500 Fast Real-Time PCR System (Applied Biosystems) for 14 cycles at 95 °C for 15 seconds and at 60 °C for 4 minutes. After the cycling, the pre-amplicons were diluted 1:5 with RNase-free water to a final volume of 25 μl. A Fluidigm 96.96 Dynamic Array chip was used to perform the qPCR assays. The chip was primed in an integrated fluidic circuit controller. After the priming, 2.75 μl of 20X TaqMan gene expression assay (Applied Biosystems) were mixed with 2.75 μl of 2X assay loading reagent (100–7611, Fluidigm) individually and loaded into the assay inlet on the chip; 2.25 μl of preamplified cDNA were mixed with 2.5 μl of TaqMan Universal PCR Master Mix (4304437, Applied Biosystems) and 0.25 μl of 20X GE sample loading reagent (100–7610, Fluidigm) and loaded into the sample inlet on the chip. The chip was returned to the integrated fluidic circuit controller for loading. After the samples and assays were loaded, the chip was placed into the BioMark System to amplify the target genes. The cycle threshold (Ct) value of each reaction on the chip was obtained with the Fluidigm RT-PCR analysis software.

### Data analysis

#### Microarray data preprocessing

Human Transcriptome Arrays contain >6.0 million distinct probes grouped into probesets that target the exonic regions of 245,349 coding and 40,914 non-coding transcripts drawn from multiple data sources (RefSeq, Ensembl, UCSC, etc.). Microarray raw gene expression data were background corrected, quantile normalized, and summarized for each of the 44,699 coding and 22,829 corresponding transcript clusters (genes) using Robust Multi-array Average (RMA)^81^ implemented in the *oligo* package^82^ using probe to transcript cluster assignments from the *hta20sttranscriptcluster.db* package of Bioconductor^83^. P-values for expression above background levels for each probeset targeting individual exons of the genes were obtained using the Affymetrix Expression Console^TM^ version 1.4. Identification of genes as protein coding was based on HTA-2_0.na36.hg19.transcript annotation provided by the manufacturer. Microarray data were deposited in the Gene Expression Omnibus at https://www.ncbi.nlm.nih.gov/geo/query/acc.cgi?acc=GSE113809.

#### Preprocessing RNA-Seq data

Paired-end RNA-Seq sequence data in *fastq* format were processed using Salmon aligner (version 0.9.1)^40^ in quasi-mapping-based mode using the *Ensembl GRCh37.75* (hg19) version of the transcriptome that included both coding and non-coding genes. Expression quantification included correction for sequence-specific biases and fragment-level GC biases to generate counts per million for each transcript scaled up to library size. Sequence count data inferred from Salmon gene abundance were imported into R using the *tximport* package^84^. Raw and processed data from RNA-Seq experiments are available at https://www.ncbi.nlm.nih.gov/geo/query/acc.cgi?acc=GSE113964.

#### Pre-processing targeted RNA-Seq data

Representation of gene-specific amplicons in amplified cDNA products was analyzed using custom gene enumeration software developed by Cellecta, Inc. With this method, only reads that align to the gene-specific amplicon are counted. The alignment uses only the 36 nucleotides started by forward primer position and 36 nucleotides region ended by reverse primer. Any reads from the middle portion of the amplicons are not counted. To determine the impact of the expression quantification procedure, the exact same procedure based on Salmon aligner^40^ was also used to generate alternative count summaries for DriverMap. Raw and processed data for DriverMap sequencing experiments are available at https://www.ncbi.nlm.nih.gov/geo/query/acc.cgi?acc=GSE114037.

#### Quantification of cell type-specific expression

Thirteen cell type-specific gene sets (3 to 27 genes each) identified in placental single-cell genomics^2^ were summarized in each sample by averaging expression data (log_2_ normalized intensity for microarrays, log_2_ normalized counts for RNA-Seq and DriverMap, and −ΔCt for qRT-PCR).

#### Differential expression analysis

Gene expression data from all four platforms (microarray, RNA-Seq, targeted expression profiling, and qRT-PCR) were analyzed to quantify the effect of gestational age and the effect of labor at term on gene expression.

The effect of gestation was assessed within subject by performing a paired analysis contrasting expression data in one sample at term (gestational age ≥37 weeks) against the two samples collected preterm (<37 weeks). This analysis was restricted to the eight women who had three longitudinal measurements. To quantify changes with gestational age, we used linear models in which the response variable was the expression of each gene, while independent variables included the gestational age as a binary variable (term vs preterm gestation) as well as a subject-specific fixed effect, hence implementing a paired (within subject) analysis.

The effect of spontaneous labor at term was evaluated by performing an unpaired analysis of expression data in samples collected at term between eight women with term labor (TIL) and eight women who delivered at term without spontaneous labor (TNL). Linear models used to assess the effect of spontaneous labor at term included the expression of each gene as response variable and the group (TIL vs TNL) as an independent variable.

Microarray gene expression data were analyzed using linear models implemented in the *limma* package^85^ of Bioconductor. qRT-PCR data were analyzed using the same type of models starting with −ΔCt values, which are surrogates of log_2_ expression normalized with respect to housekeeping genes (*ACTB*, *B2M*, *GAPDH*, *POLR2A*, *RPL37A*, and *RPLPO*).

Count data obtained for the two sequencing based platforms (RNA-Seq and DriverMap) were analyzed using negative binomial models implemented in the *DESeq. 2* package^35,86^ according to the same independent variables described for microarray and qRT-PCR data.

For all three high-throughput gene expression platforms, differences were considered significant if the false discovery rate-adjusted p-values (q-value) were <0.1, given that the transcripts were deemed expressed. The less conservative false discovery rate cut-off (10% as opposed to 5%) was selected to provide increased power to assess differences in the number of genes differentially expressed with a given phenotype by the three expression profiling methods, and also to quantify the overlap with lists of genes reported in other studies. For microarrays, transcript clusters were considered expressed if at least one probeset targeting the same transcript cluster had a detection p-value < 0.05 in at least 5 of the 32 samples. For sequencing based methods, a raw sequence count ≥5 in at least 5 of the 32 samples was required to call a gene expressed.

#### Selection of candidate genes for validation

The design of the qRT-PCR validation study was based on a preliminary analysis of the high-throughput data and aimed to include the top 10 genes identified by only one method and all genes identified by all three methods in each of the two comparisons (effect of gestational age and effect of labor). The preliminary analysis differed from the one reported above in terms of 1) criteria used to define expression above background for microarrays (using a cut-off for absolute intensity as opposed to detection p-values), 2) did not involve the exclusion of the contaminated sample for DriverMap analysis, and 3) used different differential expression criteria [nominal p < 0.005 and absolute log_2_ ratio > log_2_ (1.5)]. The assay identifiers for the target and reference genes used in the qRT-PCR experiments are shown in Table S3.

### Ethics approval and consent to participate

All patients provided written informed consent. The use of biological specimens as well as clinical data for research purposes were approved by the Institutional Review Boards of Wayne State University and NICHD.

## Electronic supplementary material

Supplementary Information

## Data Availability

The raw and summarized expression data for microarrays, RNA-Seq and targeted expression profiling by RNA-Seq are available as a Gene Expression Omnibus super series (https://www.ncbi.nlm.nih.gov/geo/query/acc.cgi?acc=GSE113966). The R analysis script and data required to reproduce the main figures and tables is available from the authors’ website at http://bioinformaticsprb.med.wayne.edu/software/.
