# Supplementary material for: Targeted expression profiling by RNA-Seq improves detection of cellular dynamics during pregnancy and identifies a role for T cells in term parturition
Source: Sci Rep. 2019 Jan 29;9:848. doi: 10.1038/s41598-018-36649-w (PMC6351599; doi:10.1038/s41598-018-36649-w)
Supplement: Supplementary file 1 — Supplementary Information [file 41598_2018_36649_MOESM1_ESM.doc]

**Supplementary Materials: Targeted expression profiling by RNA-Seq improves detection of cellular dynamics during pregnancy and identifies a role for T cells in term parturition**

Adi L. Tarca, PhD^1,2,3*^

[atarca@med.wayne.edu](mailto:atarca@med.wayne.edu)

Roberto Romero, MD, D.Med.Sci.^1,4,5,6*^

[prbchiefstaff@med.wayne.edu](mailto:prbchiefstaff@med.wayne.edu)

Zhonghui Xu, MSc^7^

[rezxu@channing.harvard.edu](mailto:rezxu@channing.harvard.edu)

Nardhy Gomez-Lopez, PhD^1,2,8^

[ngomezlo@med.wayne.edu](mailto:ngomezlo@med.wayne.edu)

Offer Erez, MD^1,2,8^

[oerez@med.wayne.edu](mailto:oerez@med.wayne.edu)

Chaur-Dong Hsu, MD^2^

[chsu@med.wayne.edu](mailto:chsu@med.wayne.edu)

Sonia S. Hassan, MD^1,2,9^

[shassan@med.wayne.edu](mailto:shassan@med.wayne.edu)

Vincent J. Carey, PhD^7^

[vincent.carey@channing.harvard.edu](mailto:vincent.carey@channing.harvard.edu)

^1^Perinatology Research Branch, Division of Obstetrics and Maternal-Fetal Medicine, Division of Intramural Research, *Eunice Kennedy Shriver* National Institute of Child Health and Human Development, National Institutes of Health, U.S. Department of Health and Human Services (NICHD/NIH/DHHS), Bethesda, Maryland, and Detroit, Michigan, USA;

^2^Department of Obstetrics and Gynecology, Wayne State University School of Medicine, Detroit, Michigan, USA;

^3^Department of Computer Science, Wayne State University College of Engineering, Detroit, Michigan, USA;

^4^Department of Obstetrics and Gynecology, University of Michigan, Ann Arbor, Michigan, USA;

^5^Department of Epidemiology and Biostatistics, Michigan State University, East Lansing, Michigan, USA;

^6^Center for Molecular Medicine and Genetics, Wayne State University, Detroit, Michigan, USA;

^7^Channing Laboratory, Department of Medicine, Brigham and Women’s Hospital and Harvard Medical School

^8^Department of Obstetrics and Gynecology. Soroka University Medical Center, School of Medicine, Faculty of Health Sciences, Ben-Gurion University of the Negev, Beersheba, Israel

^9^Department of Physiology, Wayne State University School of Medicine, Detroit, Michigan, USA.

Correspondence should be addressed to: ALT (email: [atarca@med.wayne.edu](mailto:atarca@med.wayne.edu)) or RR (email: [prbchiefstaff@med.wayne.edu](mailto:prbchiefstaff@med.wayne.edu))

**Table S1: Metadata for the 32 mRNA samples used in this study**. ID: identifier, GA: gestational age in weeks; TIL: term in labor; TNL: term not in labor; RIN: RNA integrity number.

| **Sample ID** | **Individual ID** | **GA** | **Group** | **Included in GA Analysis** | **Included in Labor Analysis** | **RIN** | **Storage (Months)** |
| --- | --- | --- | --- | --- | --- | --- | --- |
| Sample_10 | mi548 | 26 | TIL | 1 | 0 | 6.3 | 47.4 |
| Sample_11 | mi548 | 34.7 | TIL | 1 | 0 | 6.4 | 45.4 |
| Sample_12 | mi548 | 40 | TIL | 1 | 1 | 6.8 | 44.2 |
| Sample_13 | mi334 | 18.9 | TIL | 1 | 0 | 6.7 | 63 |
| Sample_14 | mi334 | 33.9 | TIL | 1 | 0 | 6.8 | 59.5 |
| Sample_15 | mi334 | 40 | TIL | 1 | 1 | 6.3 | 58.1 |
| Sample_18 | mi751 | 38.9 | TNL | 0 | 1 | 6.9 | 11.1 |
| Sample_19 | mi708 | 19.9 | TNL | 1 | 0 | 6.5 | 16.3 |
| Sample_20 | mi708 | 29.9 | TNL | 1 | 0 | 6.6 | 14 |
| Sample_21 | mi708 | 37.3 | TNL | 1 | 1 | 6.5 | 12.3 |
| Sample_22 | mi323 | 12.7 | TNL | 1 | 0 | 6.2 | 21.6 |
| Sample_23 | mi323 | 25.7 | TNL | 1 | 0 | 6.9 | 18.6 |
| Sample_24 | mi323 | 38.7 | TNL | 1 | 1 | 6.8 | 15.5 |
| Sample_25 | mi695 | 25.3 | TNL | 1 | 0 | 6.8 | 16.1 |
| Sample_26 | mi695 | 34.7 | TNL | 1 | 0 | 7.1 | 13.9 |
| Sample_27 | mi695 | 39 | TNL | 1 | 1 | 7.1 | 12.9 |
| Sample_28 | mi424 | 19.9 | TNL | 1 | 0 | 6.9 | 44.7 |
| Sample_29 | mi424 | 35.1 | TNL | 1 | 0 | 7.2 | 41.1 |
| Sample_3 | mi609 | 40.3 | TIL | 0 | 1 | 6.7 | 55.8 |
| Sample_30 | mi424 | 39.1 | TNL | 1 | 1 | 7.4 | 40.2 |
| Sample_31 | mi134 | 39.7 | TIL | 0 | 1 | 7.3 | 58.7 |
| Sample_32 | mi417 | 40 | TIL | 0 | 1 | 7.4 | 56.8 |
| Sample_34 | mi379 | 38.6 | TIL | 0 | 1 | 6.4 | 46.8 |
| Sample_36 | mi270 | 39 | TNL | 0 | 1 | 7.1 | 16.8 |
| Sample_37 | mi306 | 38.7 | TNL | 0 | 1 | 6.7 | 14.3 |
| Sample_38 | mi411 | 39.3 | TNL | 0 | 1 | 6.5 | 13.8 |
| Sample_4 | mi686 | 18.6 | TIL | 1 | 0 | 6.6 | 62.1 |
| Sample_5 | mi686 | 30.6 | TIL | 1 | 0 | 7.2 | 59.3 |
| Sample_6 | mi686 | 40.1 | TIL | 1 | 1 | 6.9 | 57.1 |
| Sample_7 | mi058 | 12.1 | TIL | 1 | 0 | 6.7 | 58.1 |
| Sample_8 | mi058 | 26.1 | TIL | 1 | 0 | 6.9 | 54.9 |
| Sample_9 | mi058 | 39.3 | TIL | 1 | 1 | 6.9 | 51.8 |

**Table S2: Differential expression with gestational age**. Positive/negative log_2_ ratios represent increase/decrease with gestation (term vs preterm). Grey highlight corresponds to significant changes based on qRT-PCR (p<0.05). The last three columns indicate whether the gene was significant according to the corresponding platform at q<0.1.

|  | **qRT-PCR** |  |  | **Significant by** | |
| --- | --- | --- | --- | --- | --- |
| **SYMBOL** | **log2 Ratio** | **p-value** | **HTA** | **RNASeq** | **DriverMap** |
| ANXA1 | 1.1 | 6.1E-07 | 1 | 1 | 1 |
| IFIT1 | 1.1 | 4.0E-06 | 1 | 1 | 1 |
| RPS24 | 1.1 | 4.7E-06 | 1 | 1 | 0 |
| HAT1 | 1.0 | 5.6E-06 | 1 | 0 | 1 |
| KLRF1 | 1.0 | 7.7E-06 | 1 | 0 | 1 |
| BCL2A1 | 1.6 | 8.6E-06 | 1 | 1 | 1 |
| POLR2K | 1.1 | 9.0E-06 | 1 | 1 | 1 |
| SKIL | 0.8 | 1.2E-05 | 1 | 1 | 1 |
| UQCRQ | 0.9 | 1.2E-05 | 0 | 0 | 1 |
| S100A8 | 1.5 | 1.7E-05 | 1 | 1 | 1 |
| CD69 | 0.9 | 2.2E-05 | 1 | 1 | 1 |
| GZMA | 0.8 | 2.3E-05 | 1 | 1 | 1 |
| RPL9 | 1.1 | 2.9E-05 | 1 | 1 | 1 |
| GIMAP2 | 0.7 | 3.5E-05 | 1 | 1 | 1 |
| AKR1C3 | 0.9 | 3.5E-05 | 0 | 0 | 1 |
| RPS27 | 0.9 | 3.8E-05 | 1 | 1 | 1 |
| AP1S2 | 0.6 | 5.0E-05 | 0 | 1 | 1 |
| NDUFS4 | 0.7 | 1.0E-04 | 0 | 0 | 1 |
| RPL21 | 1.0 | 1.1E-04 | 1 | 0 | 1 |
| IFIT5 | 0.6 | 1.2E-04 | 1 | 1 | 1 |
| RPS25 | 0.6 | 1.4E-04 | 1 | 1 | 1 |
| CHMP5 | 0.8 | 1.6E-04 | 1 | 1 | 1 |
| EEPD1 | -0.6 | 1.8E-04 | 1 | 1 | 1 |
| PSMC2 | 0.5 | 2.5E-04 | 1 | 1 | 1 |
| WBP2 | -0.7 | 2.7E-04 | 0 | 0 | 0 |
| IDI1 | 0.6 | 2.8E-04 | 1 | 1 | 1 |
| SAMD9L | 0.5 | 3.0E-04 | 1 | 1 | 1 |
| MRPL40 | 0.6 | 3.5E-04 | 0 | 0 | 1 |
| SAMD9 | 0.9 | 3.6E-04 | 1 | 1 | 1 |
| COPS2 | 0.8 | 4.2E-04 | 1 | 0 | 1 |
| APOL4 | 0.9 | 4.9E-04 | 0 | 0 | 0 |
| ERGIC2 | 0.6 | 6.2E-04 | 1 | 0 | 1 |
| HMGB2 | 0.6 | 6.4E-04 | 1 | 0 | 1 |
| SHISA4 | -0.7 | 6.6E-04 | 0 | 0 | 1 |
| RPL37 | 0.5 | 7.4E-04 | 1 | 1 | 1 |
| PIGK | 0.5 | 7.6E-04 | 1 | 1 | 1 |
| EVI2A | 0.7 | 8.2E-04 | 1 | 1 | 1 |
| ANXA3 | 0.7 | 8.4E-04 | 1 | 0 | 1 |
| SCARNA7 | 0.6 | 1.3E-03 | 1 | 1 | 0 |
| TAX1BP1 | 0.6 | 1.8E-03 | 1 | 1 | 1 |
| RPL17 | 0.5 | 2.7E-03 | 0 | 1 | 0 |
| RPL6 | 0.4 | 3.7E-03 | 1 | 1 | 1 |
| RPL17-C18orf32 | 0.5 | 4.0E-03 | 0 | 0 | 1 |
| HINT1 | 0.5 | 5.1E-03 | 0 | 1 | 1 |
| BIRC2 | 0.6 | 5.4E-03 | 0 | 0 | 1 |
| ZNF213 | -0.4 | 7.2E-03 | 0 | 0 | 0 |
| A4GALT | -0.5 | 7.9E-03 | 0 | 0 | 0 |
| MTHFD2 | 0.3 | 8.3E-03 | 1 | 1 | 1 |
| SNORD116-8 | 0.6 | 9.0E-03 | 1 | 0 | 0 |
| GNAS | -0.4 | 1.2E-02 | 0 | 0 | 0 |
| SNORD116-2 | 0.5 | 1.5E-02 | 1 | 0 | 0 |
| GZMH | 0.4 | 1.7E-02 | 0 | 0 | 1 |
| CAMSAP1 | -0.3 | 1.7E-02 | 0 | 0 | 0 |
| IGF2BP2 | -0.5 | 1.7E-02 | 0 | 0 | 0 |
| SNORA49 | 0.5 | 1.9E-02 | 1 | 1 | 0 |
| C1GALT1C1 | 0.3 | 2.0E-02 | 0 | 0 | 1 |
| SCARNA5 | 0.5 | 2.3E-02 | 1 | 1 | 0 |
| GZMB | 0.6 | 3.1E-02 | 1 | 1 | 1 |
| KLRC3 | 0.4 | 3.4E-02 | 0 | 0 | 0 |
| SNORD59B | 0.4 | 3.5E-02 | 1 | 0 | 0 |
| BACE2 | -0.4 | 3.7E-02 | 0 | 0 | 0 |
| SNORD117 | 0.4 | 4.2E-02 | 0 | 0 | 0 |
| KLRD1 | 0.4 | 5.5E-02 | 0 | 0 | 1 |
| ANK1 | -0.4 | 5.8E-02 | 0 | 0 | 0 |
| FGFBP2 | 0.4 | 5.8E-02 | 0 | 0 | 1 |
| UBE2O | -0.5 | 6.4E-02 | 0 | 0 | 0 |
| KLRC2 | 0.4 | 7.7E-02 | 0 | 0 | 1 |
| CCL4 | 0.2 | 9.2E-02 | 0 | 1 | 1 |
| PRSS30P | 0.3 | 1.0E-01 | 0 | 0 | 0 |
| IGHG3 | -0.3 | 1.0E-01 | 0 | 0 | 0 |
| RP11-166O4.5 | -0.4 | 1.0E-01 | 0 | 0 | 0 |
| SPECC1 | -0.2 | 1.7E-01 | 0 | 0 | 0 |
| MYBL1 | 0.2 | 2.2E-01 | 0 | 0 | 1 |
| WIPI2 | -0.2 | 2.2E-01 | 0 | 0 | 0 |
| SCARNA6 | 0.3 | 2.4E-01 | 0 | 0 | 0 |
| SNORD104 | 0.2 | 2.5E-01 | 0 | 0 | 0 |
| RPL26 | 0.2 | 2.8E-01 | 1 | 1 | 1 |
| H1F0 | -0.3 | 2.9E-01 | 0 | 0 | 0 |
| CADM1 | 0.1 | 3.4E-01 | 0 | 0 | 0 |
| NCOA4 | -0.1 | 4.8E-01 | 0 | 0 | 0 |
| SNORA52 | -0.1 | 5.9E-01 | 0 | 0 | 0 |
| SNORD17 | 0.1 | 5.9E-01 | 0 | 0 | 0 |
| GNLY | 0.1 | 6.4E-01 | 0 | 0 | 0 |
| SNORA74A | 0.1 | 6.9E-01 | 0 | 0 | 0 |
| HIST1H2BN | 0.0 | 9.5E-01 | 0 | 0 | 0 |
| FHAD1 | 0.0 | 9.6E-01 | 0 | 0 | 0 |

**Table S3: Differential expression with labor at term.** Positive/negative log_2_ ratios represent increase/decrease with labor (term in labor vs term not in labor). Grey highlight corresponds to significant changes by qRT-PCR (p<0.05). The last three columns indicate whether the gene was significant according to the corresponding platform at q<0.1. No genes were found significant by microarrays.

|  | **qRT-PCR** | | **Significant by** | |
| --- | --- | --- | --- | --- |
| **SYMBOL** | **log2 Ratio** | **p-value** | **RNA-Seq** | **DriverMap** |
| KLRF1 | 1.3 | 1.8E-04 | 1 | 1 |
| AKR1C3 | 1.1 | 3.7E-04 | 0 | 1 |
| KLRC2 | 2.3 | 6.2E-04 | 0 | 1 |
| H1F0 | -1.2 | 1.1E-03 | 0 | 1 |
| UBE2O | -1.0 | 1.4E-03 | 0 | 1 |
| SPECC1 | -0.8 | 2.0E-03 | 0 | 1 |
| BACE2 | -0.9 | 2.0E-03 | 0 | 1 |
| FGFBP2 | 1.4 | 2.3E-03 | 0 | 1 |
| GZMB | 1.1 | 3.3E-03 | 1 | 1 |
| KLRC3 | 1.5 | 3.5E-03 | 0 | 0 |
| GZMH | 1.5 | 4.0E-03 | 0 | 1 |
| GZMA | 1.1 | 4.3E-03 | 0 | 1 |
| CD69 | 0.9 | 4.7E-03 | 1 | 1 |
| GNLY | 1.3 | 4.8E-03 | 0 | 1 |
| IGF2BP2 | -1.1 | 6.5E-03 | 0 | 1 |
| PRSS30P | 1.3 | 6.8E-03 | 1 | 0 |
| ANK1 | -0.9 | 7.2E-03 | 0 | 1 |
| KLRD1 | 1.0 | 8.8E-03 | 0 | 1 |
| CADM1 | 1.0 | 1.0E-02 | 1 | 0 |
| A4GALT | -2.3 | 1.4E-02 | 0 | 1 |
| CCL4 | 0.8 | 1.7E-02 | 0 | 1 |
| WBP2 | -0.6 | 2.2E-02 | 0 | 1 |
| MYBL1 | 0.6 | 2.4E-02 | 0 | 1 |
| GNAS | -0.6 | 2.5E-02 | 0 | 1 |
| NCOA4 | -0.7 | 3.5E-02 | 0 | 1 |
| RPS27 | 0.7 | 3.7E-02 | 0 | 1 |
| UQCRQ | 0.7 | 3.9E-02 | 0 | 0 |
| RPS25 | 0.7 | 3.9E-02 | 0 | 0 |
| RPS24 | 0.8 | 4.1E-02 | 0 | 0 |
| SCARNA6 | 0.7 | 5.0E-02 | 0 | 0 |
| SCARNA7 | 0.6 | 5.7E-02 | 0 | 0 |
| SCARNA5 | 0.7 | 5.8E-02 | 0 | 0 |
| APOL4 | 0.8 | 5.8E-02 | 0 | 0 |
| ANXA1 | 0.6 | 6.1E-02 | 0 | 0 |
| RPL21 | 0.6 | 7.5E-02 | 0 | 0 |
| WIPI2 | -0.4 | 7.6E-02 | 0 | 1 |
| HAT1 | 0.6 | 7.9E-02 | 0 | 0 |
| BCL2A1 | 0.9 | 8.0E-02 | 0 | 0 |
| IGHG3 | -0.8 | 9.4E-02 | 1 | 0 |
| IDI1 | 0.4 | 1.0E-01 | 0 | 0 |
| RPL17 | 0.5 | 1.0E-01 | 0 | 0 |
| RPL37 | 0.4 | 1.0E-01 | 0 | 0 |
| RPL6 | 0.4 | 1.1E-01 | 0 | 0 |
| POLR2K | 0.6 | 1.1E-01 | 0 | 0 |
| MTHFD2 | 0.3 | 1.1E-01 | 0 | 0 |
| RPL9 | 0.6 | 1.2E-01 | 0 | 0 |
| S100A8 | 0.7 | 1.3E-01 | 0 | 0 |
| HMGB2 | 0.5 | 1.3E-01 | 0 | 0 |
| SNORD17 | 0.4 | 1.4E-01 | 0 | 0 |
| NDUFS4 | 0.4 | 1.4E-01 | 0 | 0 |
| HINT1 | 0.4 | 1.5E-01 | 0 | 0 |
| GIMAP2 | 0.4 | 1.5E-01 | 0 | 0 |
| ZNF213 | -0.3 | 1.6E-01 | 1 | 0 |
| ERGIC2 | 0.4 | 1.7E-01 | 0 | 0 |
| ANXA3 | 0.5 | 1.7E-01 | 0 | 0 |
| MRPL40 | 0.4 | 1.8E-01 | 0 | 0 |
| SNORA74A | 0.3 | 1.8E-01 | 0 | 0 |
| SNORD59B | 0.4 | 1.8E-01 | 0 | 0 |
| SNORD117 | 0.4 | 1.9E-01 | 0 | 0 |
| SNORA49 | 0.4 | 2.3E-01 | 0 | 0 |
| PIGK | 0.3 | 2.4E-01 | 0 | 0 |
| PSMC2 | 0.3 | 2.7E-01 | 0 | 0 |
| HIST1H2BN | -0.3 | 2.8E-01 | 1 | 0 |
| SHISA4 | -0.5 | 2.9E-01 | 0 | 0 |
| EVI2A | 0.4 | 2.9E-01 | 0 | 0 |
| IFIT1 | -0.5 | 3.3E-01 | 0 | 0 |
| SKIL | 0.2 | 3.6E-01 | 0 | 0 |
| RPL17-C18orf32 | 0.2 | 3.7E-01 | 0 | 0 |
| EEPD1 | -0.2 | 3.7E-01 | 0 | 0 |
| C1GALT1C1 | 0.2 | 4.2E-01 | 0 | 0 |
| BIRC2 | -0.2 | 5.0E-01 | 0 | 0 |
| CAMSAP1 | -0.1 | 6.0E-01 | 1 | 0 |
| FHAD1 | 0.2 | 6.2E-01 | 1 | 0 |
| SNORA52 | 0.1 | 6.4E-01 | 0 | 0 |
| SNORD116-8 | 0.1 | 6.6E-01 | 0 | 0 |
| COPS2 | 0.1 | 6.8E-01 | 0 | 0 |
| RPL26 | -0.1 | 7.2E-01 | 0 | 0 |
| RP11-166O4.5 | 0.1 | 7.3E-01 | 1 | 0 |
| SNORD104 | 0.1 | 7.3E-01 | 0 | 0 |
| IFIT5 | -0.1 | 7.9E-01 | 0 | 0 |
| SAMD9 | 0.1 | 8.5E-01 | 0 | 0 |
| CHMP5 | 0.1 | 8.6E-01 | 0 | 0 |
| TAX1BP1 | 0.0 | 9.2E-01 | 0 | 0 |
| SNORD116-2 | 0.0 | 9.3E-01 | 0 | 0 |
| SAMD9L | 0.0 | 9.6E-01 | 0 | 0 |
| AP1S2 | 0.0 | 9.6E-01 | 0 | 0 |

**Table S4: List of 86 target and 6 house-keeping genes and TaqMan™ catalog identifiers.**

| **Gene Symbol** | **TaqMan Assay ID** |
| --- | --- |
| A4GALT | Hs04962933_m1 |
| AKR1C3 | Hs00366267_m1 |
| ANK1 | Hs00986657_m1 |
| ANXA1 | Hs00167549_m1 |
| ANXA3 | Hs00971411_m1 |
| AP1S2 | Hs04999379_m1 |
| APOL4 | Hs00540930_m1 |
| BACE2 | Hs00273238_m1 |
| BCL2A1 | Hs00187845_m1 |
| BIRC2 | Hs01112284_m1 |
| C18orf32, RPL17-C18orf32 | Hs00743508_s1 |
| C1GALT1C1 | Hs00384513_m1 |
| CADM1 | Hs00942509_m1 |
| CAMSAP1 | Hs00251465_m1 |
| CCL4 | Hs99999148_m1 |
| CD69 | Hs00934033_m1 |
| CHMP5 | Hs00603789_mH |
| COPS2 | Hs00182826_m1 |
| EEPD1 | Hs00286893_m1 |
| ERGIC2 | Hs00275449_m1 |
| EVI2A | Hs00204405_m1 |
| FGFBP2 | Hs00230605_m1 |
| FHAD1 | Hs00948384_m1 |
| GIMAP2 | Hs00385026_m1 |
| GNAS | Hs00255603_m1 |
| GNLY | Hs01120727_m1 |
| GZMA | Hs00989184_m1 |
| GZMB | Hs00188051_m1 |
| GZMH | Hs00277212_m1 |
| H1F0 | Hs00271174_s1 |
| HAT1 | Hs00186320_m1 |
| HINT1 | Hs00602163_m1 |
| HIST1H2BN | Hs00534080_s1 |
| HMGB2 | Hs01127828_g1 |
| IDI1 | Hs01057440_m1 |
| IFIT1 | Hs01675197_m1 |
| IFIT5 | Hs00202721_m1 |
| IGF2BP2 | Hs01118006_m1 |
| IGHG3, IGHM | Hs00382386_m1 |
| KLRC2 | Hs02379574_g1 |
| KLRC3 | Hs01652462_m1 |
| KLRD1 | Hs00233844_m1 |
| KLRF1 | Hs01044622_m1 |
| MRPL40 | Hs00186843_m1 |
| MTHFD2 | Hs00741165_m1 |
| MYBL1 | Hs00979364_m1 |
| NCOA4 | Hs00428328_m1 |
| NDUFS4 | Hs00159589_m1 |
| PIGK | Hs00300778_m1 |
| POLR2K | Hs01562397_m1 |
| PRSS30P | Hs00698405_m1 |
| PSMC2 | Hs00739800_m1 |
| RP11-166O4.5 | AP9HJ6T |
| RPL17 | Hs00932568_m1 |
| RPL21, RPL21P28 | Hs03003806_g1 |
| RPL26 | Hs00864008_m1 |
| RPL37 | Hs03044965_g1 |
| RPL6 | Hs03044365_g1 |
| RPL9 | Hs01552541_g1 |
| RPS24 | Hs03006009_g1 |
| RPS25 | Hs01568661_g1 |
| RPS27 | Hs04185005_g1 |
| S100A8 | Hs00374263_m1 |
| SAMD9 | Hs00415836_m1 |
| SAMD9L | Hs00416109_m1 |
| SCARNA5 | Hs03298717_s1 |
| SCARNA6 | Hs03298715_s1 |
| SCARNA7 | Hs03309492_s1 |
| SHISA4 | Hs00411448_m1 |
| SKIL | Hs01045418_m1 |
| SNORA49 | Hs03298693_s1 |
| SNORA52 | Hs03297911_s1 |
| SNORA74A | Hs03298571_s1 |
| SNORD104 | Hs03309800_s1 |
| SNORD116-2 | APEPT94 |
| SNORD116-8 | APFVMU2 |
| SNORD117 | Hs03299042_s1 |
| SNORD17 | Hs03298746_s1 |
| SNORD59B | Hs03309513_s1 |
| SPECC1 | Hs00411658_m1 |
| TAX1BP1 | Hs00195718_m1 |
| UBE2O | Hs00222904_m1 |
| UQCRQ | Hs00429571_g1 |
| WBP2 | Hs00600857_m1 |
| WIPI2 | Hs00255379_m1 |
| ZNF213 | Hs00383373_m1 |
| ACTB | Hs99999903_m1 |
| B2M | Hs00984230_m1 |
| GAPDH | Hs99999905-m1 |
| POLR2A | Hs00172187_m1 |
| RPL37A | Hs01102345_m1 |
| RPLPO | Hs99999902_m1 |

**Figure S1: Average qRT-PCR cycle threshold (CT) of house-keeping genes as a function of the duration of storage.** The figure shows the average cycle threshold (CT) values over house-keeping genes (ACTB, B2M, GAPDH, POLR2A, RPL37A, RPLPO) used to normalize qRT-PCR experiment data as a function of the duration of sample storage from collection to mRNA extraction.

**Figure S2: Ranks of genes tested by qRT-PCR for changes with gestational age.** The figure shows the ranks of the genes that were tested by qRT-PCR and were detected and found positive for changes with gestation according to each transcriptomics platform (see also **Table S2**). The ranks of genes among the differentially expressed lists (sorted by p-value) are normalized by the size of the list of significant genes generated by each method. Genes at the bottom of the graph have smallest p-values.

**Figure S3: Ranks of genes tested by qRT-PCR for changes with labor at term.** The figure shows the ranks of the genes that were tested by qRT-PCR and were detected and found positive for changes with labor at term according to each transcriptomics platform (see also **Table S3**). The ranks of genes among the differentially expressed lists (sorted by p-value) are normalized by the size of the list of significant genes generated by each method. Genes at the bottom of the graph have smallest p-values.

**Figure S4**: UpSet plot of genes differentially expressed with gestation using three transcriptomics platforms. Unlike in Figure 1, where the adjusted p-value (q-value) <0.1 cut-off was used, in this analysis, selection of genes was based on q<0.05. The horizontal bars show the number of differentially expressed genes identified by each method, while the vertical bars display the size of sets of genes identified by only one method and the intersection sets.

**Figure S5**: UpSet plot of genes differentially expressed with gestation using three transcriptomics platforms. Unlike in Figure 1, only genes detected present on all three platforms were considered and significance of gene expression changes was inferred based on adjusted p-value (q-value) <0.1. The horizontal bars show the number of differentially expressed genes identified by each method, while the vertical bars display the size of sets of genes identified by only one method and the intersection sets.
